# Coronagraben in Switzerland: culture and social distancing in times of COVID-19

**DOI:** 10.1007/s00148-021-00865-y

**Published:** 2021-07-27

**Authors:** Neha Deopa, Piergiuseppe Fortunato

**Affiliations:** 1 The Graduate Institute of International and Development Studies, Geneva, Switzerland; 2 United Nations Conference on Trade and Development (UNCTAD), Geneva, Switzerland

**Keywords:** COVID-19, Culture, Social distancing, Trust, Redistribution, Mobility, H12, Z1, D91

## Abstract

Social distancing measures help contain the spread of COVID-19, but actual compliance has varied substantially across space and time. We ask whether cultural differences underlie this heterogeneity using mobility data across Switzerland between February and December 2020. We find that German-speaking cantons decreased their mobility for non-essential activities significantly less than French-speaking cantons. However, we find no such significant differences for bilingual cantons. Contrary to the evidence in the literature, we find that within the Swiss context, high trusting areas exhibited a smaller decline in mobility. Additionally, cantons supporting a limited role of the state in matters of welfare also experienced a smaller reduction in mobility.

## Introduction

Following the initial outbreak in China, in early January 2020, COVID-19 began rapidly spreading across all regions of the world, achieving a pandemic status. Flattening the contagion curve became a priority in many countries in an attempt to reduce the load on the healthcare system and the overall mortality rate. Several countries enacted shelter-in-place and social distancing measures to reduce interpersonal contact and mobility in order to curb transmission of the virus. This was often implemented through massive “stay at home” media campaigns aimed at altering citizens’ habits. While the health measures enacted have been, by and large, homogeneous across countries, compliance with these rules varied widely with local context. In the absence of perfect enforcement capacity by states, cultural attitudes and behavioral norms, which typically vary from country to country, can make an important difference and explain deviations in voluntary compliance. This is all the more true when it comes to individual mobility decisions, which entail a delicate trade-off between the chance of contracting (or diffusing) a disease and the economic (and individual well being) costs associated with significant alterations of daily activities.

Recent literature cites several key factors that may underlie individual compliance with social distancing measures.^1^ Cultural values and social contact patterns have previously been shown to be a crucial factor behind the transmission and risk of disease (Dressler 2004; Borg 2014). Can there be a role for cultural forces in the spread of pandemics? We study how cultural values and beliefs may play a role in the evolution of individual mobility under COVID-19 measures. Examining various dimensions of culture, we focus our analysis on Switzerland, which provides a unique case study due to its native language groups that are shared with adjoining countries. These distinct linguistic geographical areas have deep historical roots and are associated with specific cultural traits. An example that highlights this is the colloquial name for the border between the French- and German-speaking region, called Röstigraben. *Rösti* refers to a hashed potato dish which originated in the canton of Bern and is typical of Swiss German cuisine, and *Graben* means a trench or division. The intensity of the COVID-19 pandemic has varied substantially between the Swiss regions, and the divide around the spread of the virus has been defined by some observers as a *Coronagraben* in reference to the cultural border.

We add to the literature by focusing on a set of cultural dimensions and mechanisms that may have shaped actual adherence with social distancing in Switzerland between February 15 and December 31, 2020. More precisely, we first examine the relationship between the change in human mobility for retail and recreational activities, capturing compliance, and language as a proxy for culture. To further investigate this link, we then explore the role of a specific set of cultural traits associated with the linguistic background—trust in others and preferences for re-distributive policies. As we are concerned with the entire course of the pandemic in 2020, our analysis focuses on four important dates. The first is March 16, when the Swiss Federal Council declared an “extraordinary situation” instituting a ban on all private and public events and closing venues such as restaurants and bars. This was the only national intervention of this scale in Switzerland and was uniform across cantons. The second is April 27, which saw a gradual easing of measures, followed by June 22, when all mobility restrictions were suspended. The final date is October 19, when the second wave was in progress and the cantons had begun to implement their own restrictions. We discuss the differences between the first and second lockdowns in Section 3.1, especially highlighting the fact that the latter was heterogeneous across regions and there was a lack of comparable intervention.

Isolating the effect of culture is difficult due to potential local confounding factors that may also have an independent effect on compliance and mobility. To tackle this, we control for cantonal demographic, health, and socio-economic characteristics prior to the pandemic and interact them with time dummies to address their effect on the evolution of mobility patterns beyond that of culture. In our empirical model, in addition to canton and day fixed effects, we also include region-week fixed effects to capture region-specific non-linear time trends. We also control for potentially varying transmission of information between linguistic areas due to different degrees of exposure to neighbouring countries by including the number of cross-border workers based on their country of residence (France, Germany, and Italy). Finally, to emphasize that the differential mobility response is not only due to varying pandemic exposure but also cultural differences, we include the daily new cases and deaths per capita.

Using this approach, we find that in the first nationally implemented lockdown, cantons in the German linguistic region reduced their mobility by 7 percentage points *less* than the French-speaking cantons, suggesting a lower compliance with federal policies of social distancing and isolation. These differences disappeared over the subsequent months when lockdown policies were relaxed. However, the second wave of the pandemic saw them resurfacing with a similar 7 percentage points difference, although this can be in part attributable to the lack of a homogeneous federal intervention and regionally varied lockdown approach. On the contrary, for the bilingual cantons that provide an interesting middle ground between two extremes, we did not observe any strongly significant differences. Upon further analysis of distinct cultural traits we find surprising results that are at odds with the current literature on trust and compliance: mobility decline was less pronounced in high trusting areas. For cantons in the top quartile of the trust distribution, the reduction in mobility during the federal lockdown was *smaller* by 7 percentage points, and these differences continued into the ensuing phase of easing measures. Similarly, we also find that cantons with a low preference for redistribution, reflecting an anti-state stance, exhibited about a 6 percentage points *smaller* decline than other cantons during the national lockdown.

We interpret our results within the context of Switzerland, where the German linguistic region is characterized by high levels of generalized trust towards others and individualistic values. A combination of these cultural traits alter the trade-off behind individual decision on mobility. Reducing mobility becomes less relevant as an instrument to reduce the probability of contracting (or diffusing) the disease if one believes that other individuals in society will respect, among other things, physical distance and other infection prevention and control (IPC) norms, thus making mobility reduction less relevant. In a sense, physical distancing replaces social distancing. Moreover, in these cantons, reducing individual mobility due to government-imposed social distancing measures could be perceived as a sacrifice of a taller order than in more collectivist regions. Our results stress the fact that the same cultural traits can evoke varying responses under a crises such as a pandemic and therefore understanding the country specific context is crucial to policy implementation.

Our work contributes to a growing body of studies linking cultural variables, social distancing, and the spread of COVID-19 (Durante et al. 2021; Barrios et al. 2021; Borgonovi and Andrieu 2020; Brodeur et al. 2021; Bargain and Aminjonov 2020). Our results build on these papers and encapsulate some of their findings while also showing the distinctiveness of the Swiss context. Mazzonna (2020) similarly utilizes the cultural variation in Switzerland to see its effect on the spread of COVID-19, highlighting the role of elderly demographic in its growth. Complementing his results, we focus on the role of interpersonal trust and re-distributive preferences in explaining the differential compliance during the pandemic and find our results to be consistent with one another.

The remainder of the paper is organized as follows. Section 2 presents our conceptual framework, discussing the cultural differences in Switzerland. Section 3 gives a background on the COVID-19 emergency in the country and describes the data used for our analysis. Section 4 presents our empirical and identification strategy and Section 5 discusses the results. Lastly, Section 6 provides an insight into our results and concludes.

## Culture and its dimensions

We first clarify what we mean by *culture*. We follow the definition proposed by Guiso et al. (2006), where *culture* is defined as a set of “customary beliefs and values that ethnic, religious, and social groups transmit fairly unchanged from generation to generation”. We focus on language as a proxy for culture and further look at two specific dimensions or traits and explain their place in the context of Switzerland.

There is a large literature linking culture and language which builds on *The Sapir–Whorf hypothesis* emphasizing that the language one speaks influences the way one perceives the world. This hypothesis is a culmination of several early contributions by anthropologists that explored this link and whose work on cultural relativism further highlighted that language and culture were interdependent.^2^ Several studies have shown that an examination of cultural groups can be engaged by language since it has an impact on identity, values, attitudes and behaviour. More recent works of economists such as Bisin and Verdier (2011) and Ginsburgh and Weber (2020) show that the notion of a common native language is inextricably linked with cultural proximity. This goes beyond language proficiency and ability to speak and in fact captures the vertical and horizontal transmission of values.

### Generalized trust

One of the most commonly defined cultural trait is generalized trust towards others, the beliefs held about others’ trustworthiness. Alesina and La Ferrara (2002) hypothesize that this belief is a moral or cultural attitude and is positively correlated with individual characteristics such as the level and type of education received and occurrence of recent misfortunes. They also show the importance of community characteristics such as high income inequality which often leads to low interpersonal trust. From the early work of Arrow (1972), who recognized the importance of mutual trust in commercial and non-commercial transactions, the relation between generalized trust and economic development is well established (Algan and Cahuc 2014; Butler et al. 2016). It is important to note that this differs from the concept of trust in institutions, which may simply be capturing the efficiency or corruption of the government in power.

### Preferences for redistribution

Alesina and Giuliano (2011) and Alesina and Giuliano (2015) define preferences for redistribution as a situation in which one agent also cares about the utility of somebody else. They reject the notion of these preferences being unpredictable “social noise” and highlight the role of culture as an important determinant. Views on inequality and redistribution emphasize both the *value* and *belief* component of culture. Different cultures may have distinct approaches in contrasting the merits of equality versus individualism and luck versus hard work. Moreover, an individual’s predisposition to support a welfare state may also be determined by cultural traits such as perception of poverty and fairness. These values and attitudes are significantly persistent and tend to remain fairly stable over time and generations (Luttmer and Singhal 2011; Li and Liu 2019).

### Why Switzerland?

Switzerland provides an excellent case study where language is a very apt proxy for culture (Büchi 2001). There are seven greater regions containing twenty-six cantons and there are four official languages having equal status in law - German, French, Italian and Romansh. According to the 2000 census, German is spoken by 63.7% of the population, French by 20.4%, Italian by 6.5%, and Romansh by 0.5%. Three cantons—Valais, Fribourg, and Berne—are bilingual (French, German); and one canton—Graubünden—is officially trilingual (German, Romansh, Italian). From the remaining, seventeen are German speaking, four French speaking, and one Italian speaking.

**Fig. 1:**
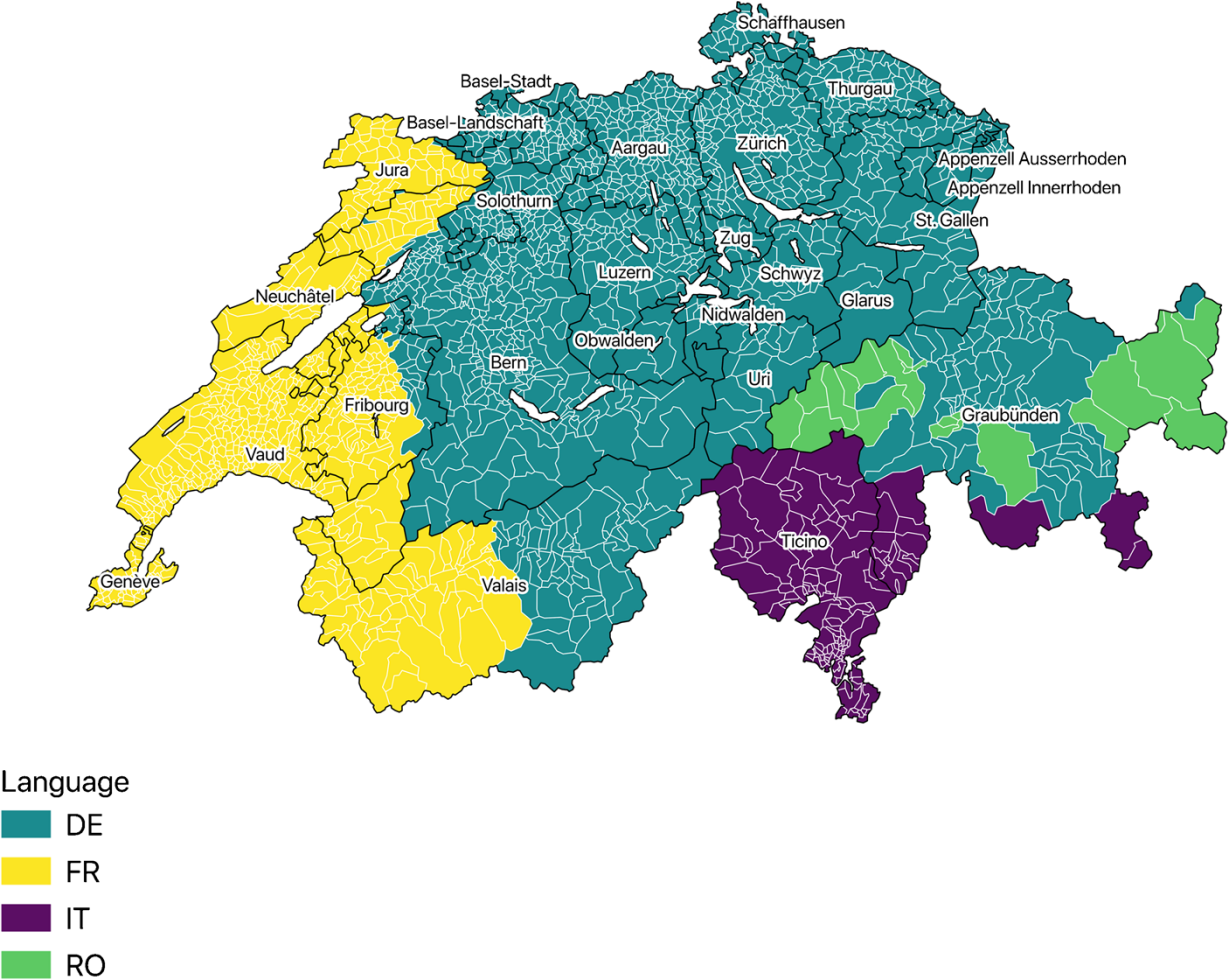
Language borders. DE: German FR: French IT: Italian RO: Romansh. The black lines represent cantonal boundaries and the white lines represent the municipal boundaries

Looking at Fig. 1, we observe that there are geographically distinct linguistic regions and these language borders in fact have deep historical roots. For instance with the exception of few minor movements, the early historical development of the German-French and German-Italian language boundaries has been relatively stable since AD 1100. Historically, the border of the canton Valais traced along the border of the Roman-Catholic Diocese of Sion and most of the canton Graubünden was once part of a Roman province called Raetia, resulting in multilingualism. The linguistic borders are a measure of cultural values and beliefs manifested by means of differences in native languages. Therefore, these explicit language regions can be thought of as pockets of different cultures and the *Röstigraben* exemplifies this fact. The language frontier manifests itself through different preferences in many aspects of political and economic decision-making thus providing an ideal context to study the effects of culture.^3^

## Background and data

### COVID-19 in Switzerland

The first case of COVID-19 in Switzerland was confirmed on 25 February 2020: a 70-year-old man tested positive in Ticino, followed by a second case on February 26 in Geneva. Due to its proximity to Lombardia, Ticino took early restrictive measures while the only rule imposed on the remaining cantons was a relatively moderate step taken by the federal government—to raise the alert level to “special situation” by banning events with more than 1000 people.^4^ However, by mid March, the country was particularly affected by the epidemic; the increase in confirmed cases accelerated with the reproductive number oscillating between 1.5 and 2 (Sciré et al. 2020). With more than 2600 people infected, there was a need to mobilise up to 8000 members of the military to help contain the rapid spread of the disease, representing the largest army mobilisation since the Second World War. This was the turning point for Switzerland and on March 16, the Swiss Federal Council declared an “extraordinary situation”, instituting a ban on all private and public events and closing restaurants, bars, leisure facilities, and shops apart from grocery stores and pharmacies. Although the government did not refer to this as a lockdown, an “extraordinary situation” is the most severe status contemplated under the law and equates to a state of emergency;^5^ therefore, for the purposes of the rest of the paper, we refer to this as the *national lockdown*.

**Fig. 2:**
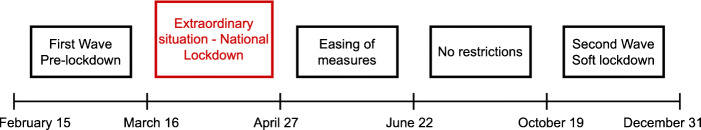
Phases of the COVID-19 pandemic in Switzerland in 2020

The first stage of relaxing the restrictions began on April 27 and the progressive easing of measures continued up until June 22. Following a widespread plateauing of cases (and incidence), the summer months saw the federal government order a full suspension of mobility restrictions, while the pandemic remained under control as in the rest of Europe. However, by early October, the cases began to surge indicating the arrival of the second wave. On November 2, there were more than 1060 cases per 100,000 inhabitants over a 14-day period. Switzerland became one of the hardest-hit countries and the contrast with its neighbours was striking: five times higher than Germany, and two times higher than Italy. Despite this, the Federal Council decided not to intervene to the extent of the first wave and left the cantons in charge of implementing their measures. This resulted in large inconsistencies between cantonal responses. For example, while Jura, Fribourg, Vaud, Neuchâtel, and Geneva implemented strict restrictions on bars, restaurants, and festivals, in the neighbouring German-speaking cantons, many cafes, leisure, and entertainment venues continued to remain open. Therefore, the second wave saw a heterogeneous response compared to the national intervention in March.^6^ Figure 2 shows a timeline of these events and the five periods which are the focus of our empirical analysis.^7^

#### Coronagraben?

**Fig. 3:**
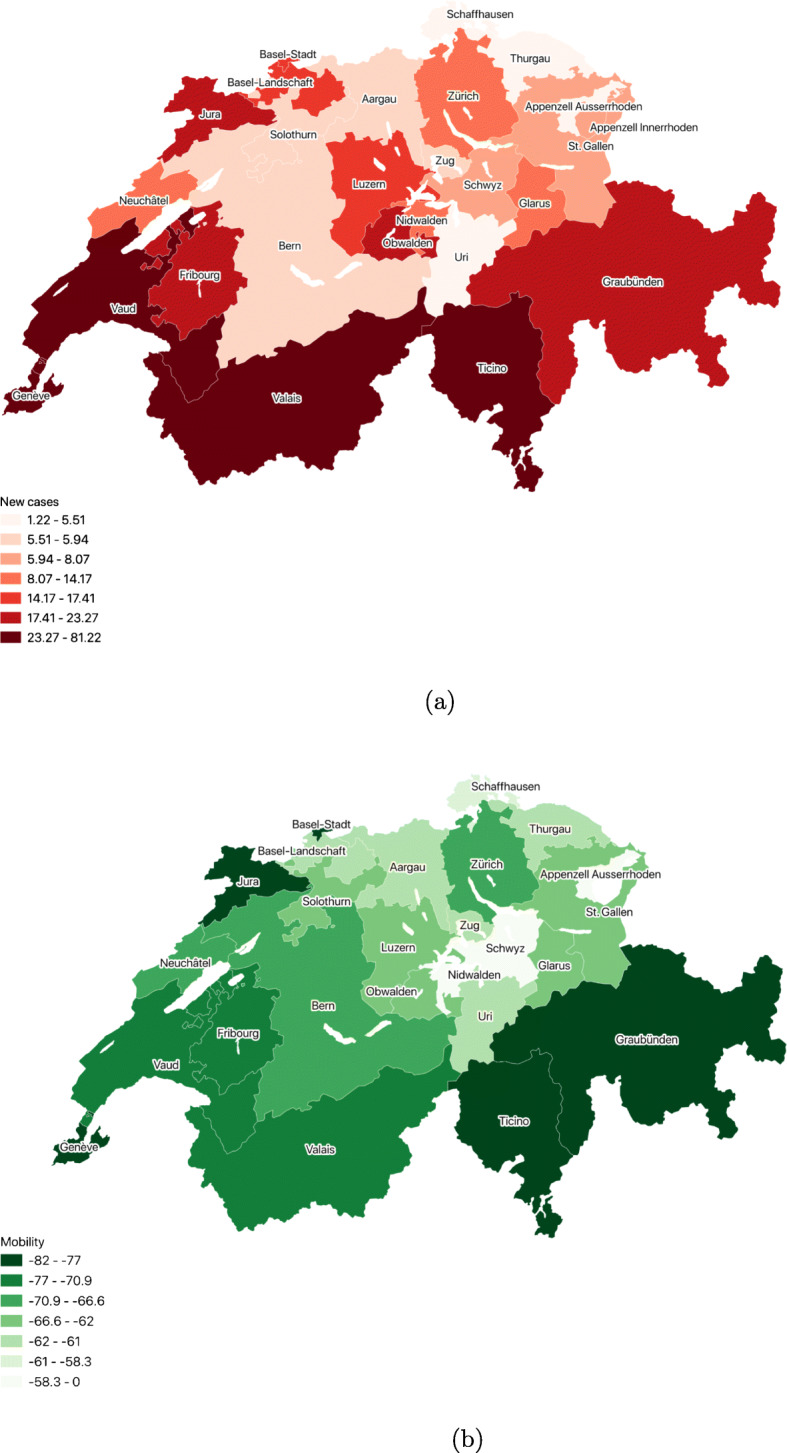
Cantonal distribution of COVID-19 cases and mobility patterns on March 27, 2020. (a) New COVID-19 cases per 100,000 inhabitants. (b) Change in mobility: Retail & Recreation

The intensity of the COVID-19 pandemic has varied substantially in the country. This is especially true for the first wave when an invisible border divided Switzerland during the crisis: the French-and Italian-speaking parts had been significantly more affected than the German-speaking areas. This linguistic divide around the spread of the virus has been defined as *Coronagraben* in reference to the cultural and political Röstigraben. This is evident in Fig. 3a where the variation in pandemic exposure is consistent with the language borders seen in Fig. 1. Correspondingly Fig. 3b maps the mobility patterns for non-essential activities across cantons and one can easily observe that the reduction in mobility was higher in the French- and Italian-speaking parts of Switzerland as compared to the German-speaking cantons. Although the second wave did not display such stark differences, regional variations continued to dominate the country, with the Swiss Romandy (Vaud, Neuchatel, Geneva, Jura Fribourg and Valais) being a COVID-19 hotspot in October and November (Fig. 7). Interestingly the *Coronagraben* was also observed within bilingual cantons, for instance in Fribourg the two German-speaking regions of the canton had the lowest incidence compared to the rest.^8^

### Data

#### Social distancing

We use Google COVID-19 Community Mobility Reports, which are created with aggregated, anonymized sets of data from users who have turned on location history.^9^ The data measures how visits to (or time spent in) different types of location change over time compared to a baseline period, which is the median value for the corresponding day of the week during January 3–February 6, 2020. The reports chart mobility trends over time by geography, across different categories of places such as retail and recreation, groceries and pharmacies, parks, transit stations, workplaces, and residential. We use the community mobility measure “Retail and Recreation” between February 15 and December 31. It is the percent change between a given day and the baseline, spent in places like restaurants, cafes, shopping centers, theme parks, museums, libraries, and movie theaters. The mobility trends of this measure capture the behavioural response to the magnitude of the pandemic and the compliance with the government measures, which may in effect vary with cultural traits.

#### Culture

Using language as a proxy for culture, we categorize each canton as (i) French, (ii) German, and (iii) Bilingual.^10^ For our main analysis, we drop Ticino as it is the only Italian-speaking canton in Switzerland, thus limiting our scope to the remaining twenty-five cantons. However, as a robustness check, we replicate our main results by including Ticino in our sample. We also use an alternative and continuous measure of language: percentage of the permanent resident population with German (or Swiss German) as their main language^11^. Data regarding official cantonal language and distribution is available on the website of the Swiss Federal Statistical Office (FSO). To measure cultural dimensions, we use the Swiss Household Panel (SHP) which is a longitudinal survey of a random sample of private households whose members represent the non-institutional population resident in Switzerland (Voorpostel et al. 2020). The principal aim of SHP is to observe social change, dynamics of living conditions, and social representations in the population.

To assess generalized trust towards others, we use wave 20 which covers the year 2018. The survey elicits beliefs by asking—*Would you say that most people can be trusted or that you can’t be too careful in dealing with people, if 0 means “Can’t be too careful” and 10 means “Most people can be trusted”?* Averaging the intensity of trust across cantons, we classify a canton as “High” trust if is above the 75th percentile. Correspondingly, to capture views on preferences for redistribution, we use wave 19 covering the year 2017.^12^ The survey asks individual opinion on the direction of federal social expenditure—*Are you in favour of a diminution or in favour of an increase of the Confederation social spending?* Using the share of respondents in each canton who are in favour of a diminution, we classify cantons as “High diminution” if they fall in the top quartile.

#### Other variables

To distinguish the effect of culture from other factors, we also include a set of social, economic and health controls at the cantonal level. Accounting for COVID-19 exposure and infection risk, we control for the number of new cases and deaths per 100,000 inhabitants, reported on the day before. To capture the quality of the health system and vulnerability to the pandemic, we use data on the number of hospital beds per 1000 inhabitants and the share of population older than 65, representing the at-risk individuals. Our specification also includes trust in federal institutions, population density, share of urban population, GDP per capita, share of population that has completed tertiary education and log of total population. Switzerland’s economy relies heavily on cross-border workers—known as frontaliers in French, Grenzgänger in German, and frontalieri in Italian. They make up about 6% of the country’s total workforce and in the secondary sector, one in ten employed workers is a cross-border worker (9.7%), with the corresponding share of 5.4% in the tertiary sector.^13^ Even during the national lockdown, the government ensured they were allowed to enter. Therefore, we control for this by using canton-quarterly data on the number of cross-border workers per 100,000 inhabitants, based on their country of residence: France, Germany, and Italy. The data on daily COVID-19 statistics is taken from the website corona-data.ch, which uses official information communicated by the cantons and Federal Office of Public Health. The remaining data is publicly available on the FSO website.

Table 1 provides summary statistics for our variables of interest. The average retail and recreation related mobility in Switzerland was about 25% lower relative to the pre-pandemic reference period. Additionally, during our sample period, Switzerland reported an average of 16.7 new cases and 0.3 new deaths per 100,000 inhabitants. However, as seen in Figs. 3 and 7, these value does not reveal the underlying variation in pandemic exposure and compliance to social distancing across cantons. We observe that in our sample the cantonal average trust in others is around 6.5, and the mean share in favour of a decrease in social spending is around 27%. Figure 8 provides a spatial distribution of these traits across cantons.

**Table 1:** Summary statistics

Variable	Mean	Median	Std. Dev.	Min	Max
Retail & Recreation	− 24.96	− 16.00	26.63	− 93.00	70.00
New cases per 100,000	16.68	2.21	31.45	0.00	283.31
New deaths per 100,000	0.30	0.00	0.85	0.00	18.58
French cross-border	2380.61	60.53	4593.16	4.09	18,092.69
workers per 100,000					
German cross-border	980.50	122.63	2007.44	5.29	8240.59
workers per 100,000					
Italian cross-border	187.01	16.10	682.34	0.00	3586.46
workers per 100,000					
Trust in others	6.49	6.60	0.38	5.78	7.30
Trust in institutions	6.32	6.28	0.20	5.97	6.89
Share in favour of diminution	26.97	27.12	4.84	15.62	36.36
Share of urban population	76.24	82.00	22.24	0.00	100.00
Share of 65+	18.93	19.10	1.72	15.70	21.90
Hospital beds per 1000	4.19	3.90	2.07	1.30	10.90
GDP per capita	82,209.42	69,860.00	33,578.57	54,291.00	203,967.00
Population	340,822.38	199,021.00	35,7910.27	16,128.00	1,539,275.00
Share of tertiary education	31.97	30.90	6.17	22.80	45.30
Share of German	68.44	86.90	32.71	4.50	93.80
as main language					
Population density	550.92	246.30	1069.96	28.00	5300.20

Our sample period of analyses is from February 15–December 31, 2020. Data for the mobility measure: Retail & Recreation is from Google COVID-19 Community Mobility Report. Trust measures and preferences for redistribution are from the Swiss Household Panel (SHP) wave 20 and 19 respectively. The additional demographic, health and socio-economic variables have been obtained from the Swiss Federal Statistical Office’s website

## Empirical strategy

### Graphical evidence

**Fig. 4:**
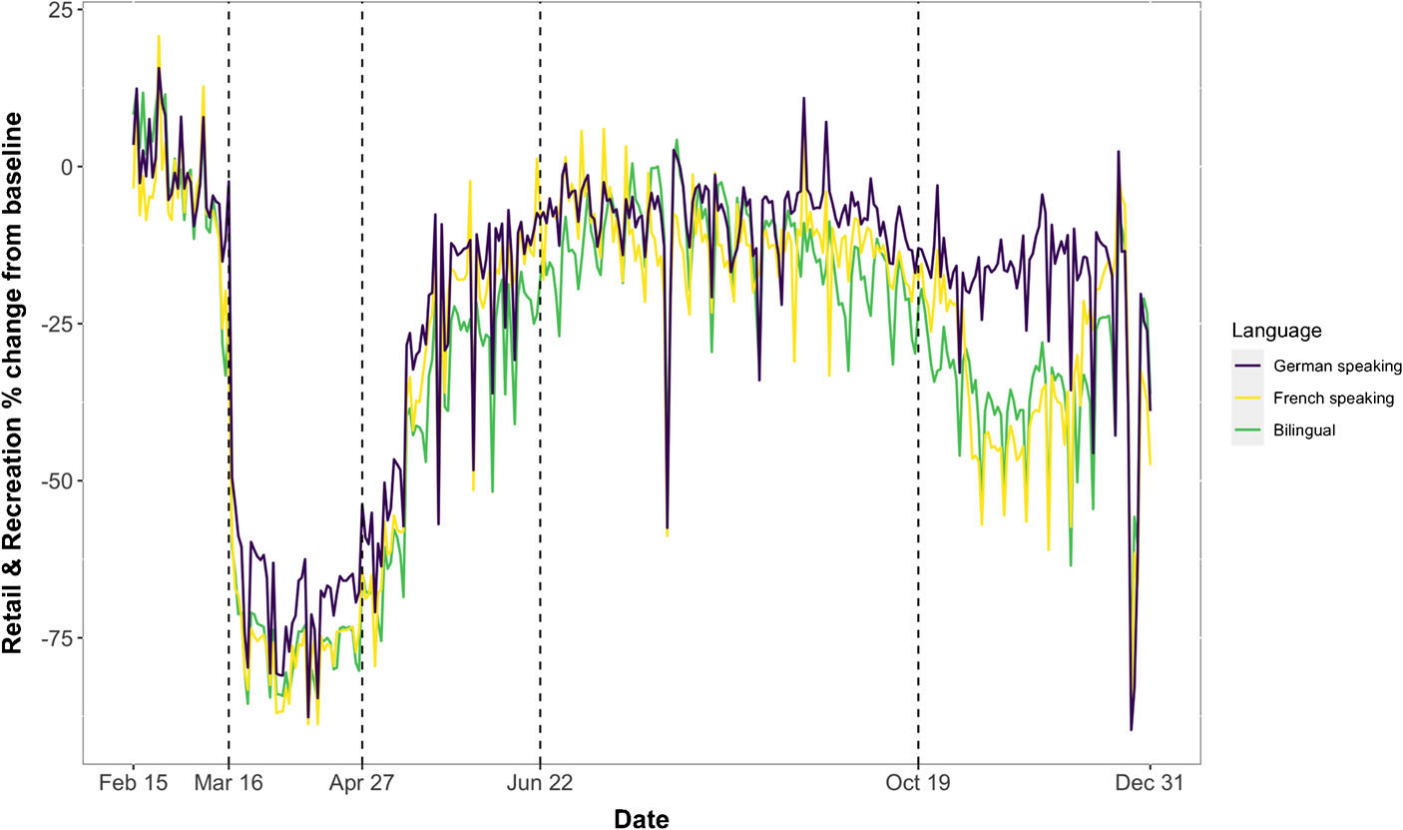
Evolution of daily mobility measure (Retail & Recreation) across the linguistic regions. Dashed lines indicate the different phases in our sample

Figures 4 and 9 visualize the relationship between mobility and linguistic regions using raw data. Figure 4 shows the evolution of daily percentage change in mobility for retail and recreation in cantons which are bilingual, French speaking and German speaking. Using the French-speaking cantons as our reference, Fig. 9 shows how the difference in mobility between the three linguistic groups evolved over time. In the weeks prior to March 16, cantons in all three linguistic regions displayed approximately similar mobility patterns. However, after the government declared an “extraordinary situation” one can distinctly observe a divergence. During the national lockdown there was a marked drop in mobility across cantons; however, the decline was notably smaller for German-speaking cantons as compared to French and bilingual. In the next two phases, the difference between French and German becomes less significant while the bilingual cantons continue to diverge often displaying a higher mobility reduction than the the rest. The final phase, which saw the rise of the second wave and the implementation of a softer and regionally heterogeneous lockdown, once again highlighted the German-speaking regions disparate response to the COVID-19 measures. To test more formally for the casual effect of culture on mobility, we discuss our econometric specification in the next section.

### Econometric model

To identify the effect of culture on social distancing, we estimate the following:

1 $$ \begin{array}{@{}rcl@{}} m_{ct} &=& \beta \text{Language}_{c} \times \textbf{D}_{t} + \delta_{1} \text{New cases}_{c,t-1} + \delta_{2} \text{New deaths}_{c,t-1} + \\ & & \gamma \textbf{Z}_{c} \times \textbf{D}_{t} + \lambda \textbf{X}_{ct} + \alpha_{c} + \theta_{t} + \phi_{rw} + \epsilon_{ct} \end{array} $$

*m*_*ct*_ is the daily Google mobility measure: “Retail and Recreation” for a given canton *c* on day *t*. Language_*c*_ is a categorical variable with cantons being classified as: Bilingual, German and French. **D**_*t*_ is a vector of time dummies indicating the five phases of the pandemic as seen in Fig. 2: (i) February 15–March 16: pre-lockdown; (ii) March 16–April 27: national lockdown; (iii) April 27–June 22: easing of measures; (iv) June 22–October 19: no restrictions; and (v) October 19–December 31: soft lockdown. Our main coefficient of interest is *β* on the interaction between Language_*c*_ and **D**_*t*_. This captures the differential evolution of mobility in cantons with different languages, as a proxy for culture, over the different phases of the pandemic.

New cases_*c*,*t*− 1_ and New deaths_*c*,*t*− 1_ represent the new COVID-19 cases and deaths per 100,000 reported in the canton up until day *t* − 1 and capture the degree of exposure and urgency to comply with social distancing measures. To isolate the effect of culture and to control for factors that maybe correlated with it and may affect the change in mobility we include **Z**_*c*_ ×**D**_*t*_ which are the interactions between the time dummies and health, demographic and socio-economic controls; and **X**_*ct*_ which is a vector of French, German, and Italian cross-border workers per capita for each canton. The specification includes a rich set of fixed-effects: *𝜃*_*t*_ daily fixed effects to account for common time trends such as the information available to all citizens affecting the common evolution of mobility. The canton fixed effects *α*_*c*_ absorb all differences in the mobility measure across cantons due to time-invariant characteristics. Finally, to further strengthen the identification, we also include *ϕ*_*rw*_ region×week fixed effects. These control for non-linear time trends specific to each of the regions, capturing weekly regional variation in compliance measures through the sample period.

The identifying assumption for Eq. 1 comes from the fact that after controlling for canton observable and unobservable time invariant characteristics, daily changes in mobility at the national level, region-specific economic, social, and policy changes over time and the severity of the pandemic at the cantonal level: the differential change in mobility in bilingual, German- and French-speaking cantons is unrelated to factors other than the ones explicitly controlled for. It is important to note that our aim is to identify how culture may have played a role in compliance and social distancing only during the national lockdown as it was homogeneous across cantons. While the remaining phases provide an interesting insight into how mobility patterns and social distancing behaviour unfolded, however, as their implementation was not uniform and elements of Swiss federalism came into play, we believe they are indicative at best. Throughout the paper, standard errors are cluster bootstrapped at the canton level.

To further examine the role of distinct cultural dimensions, we estimate:

2a $$ \begin{array}{@{}rcl@{}} m_{ct} &=& \tilde{\beta} \text{High trust}_{c} \times \textbf{D}_{t} + \delta_{1} \text{New cases}_{c,t-1} + \delta_{2} \text{New deaths}_{c,t-1} + \\ && \gamma \textbf{Z}_{c} \times \textbf{D}_{t} + \lambda \textbf{X}_{ct} + \alpha_{c} + \theta_{t} + \phi_{rw} + \epsilon_{ct}  \end{array} $$

2b $$ \begin{array}{@{}rcl@{}} m_{ct} &=& \overset{\approx}{\beta} \text{High diminution}_{c} \times \textbf{D}_{t} + \delta_{1} \text{New cases}_{c,t-1} + \delta_{2} \text{New deaths}_{c,t-1} + \\ && \gamma \textbf{Z}_{c} \times \textbf{D}_{t} + \lambda \textbf{X}_{ct} + \alpha_{c} + \theta_{t} + \phi_{rw} + \epsilon_{ct} \end{array} $$

In Eq. 2a, we explore the trait of generalized trust towards others where High trust_*c*_ is an indicator variable that takes on a value of one if the canton is above the 75th percentile of trust distribution and zero otherwise. Similarly, (2b) investigates preferences for redistribution where High diminution_*c*_ takes on a value of one if the cantonal share of respondents in favour of diminution of federal social expenditure is in the top quartile and zero otherwise.

## Results and discussion

Table 2 shows the main results from specification (1). In the first column, controlling for canton and day fixed effects, as a baseline we explore how the exposure to the pandemic itself may have influenced mobility. Not surprisingly, we find that the number of new COVID cases and deaths per capita, reported the day before, are significantly associated with reduced mobility. From column (2) onward, we introduce our main interaction terms between language and phase time dummies. We find that all models, including the most rigorous specification in column (4), convey that during the national lockdown the mobility in German-speaking region decreased *less* than the French-speaking region by around 7 percentage points: suggesting that the German-speaking cantons displayed lower compliance with federal policies of social distancing and isolation. On the contrary, during this phase, the drop in mobility for bilingual cantons did not differ significantly from its French counterpart. These results are consistent with the visual inspection of Figs. 4 and 9a.

**Table 2:** Main results

	Dependent variable:			
	Retail & Recreation			
	(1)	(2)	(3)	(4)
New cases per capita	− 0.042^∗∗∗^	− 0.025^∗∗∗^	− 0.024^∗∗∗^	− 0.022^∗∗∗^
	(0.010)	(0.007)	(0.007)	(0.007)
New deaths per capita	− 1.822^∗∗∗^	− 1.490^∗∗∗^	− 1.682^∗∗∗^	− 0.584^∗∗^
	(0.540)	(0.505)	(0.447)	(0.285)
Bilingual x national lockdown		− 1.683	1.599	1.093
		(3.490)	(3.818)	(2.823)
German x national lockdown		5.857^∗∗^	7.150^∗∗^	7.189^∗∗^
		(2.606)	(3.104)	(3.661)
Bilingual x easing of measures		− 8.039	− 3.296	− 3.939^∗^
		(5.193)	(3.852)	(2.129)
German x easing of measures		2.567	4.552^∗∗^	3.468
		(3.008)	(2.178)	(4.679)
Bilingual x no restrictions		− 4.021	0.783	0.064
		(4.239)	(4.217)	(3.301)
German x no restrictions		2.269	5.738	3.453
		(3.346)	(3.550)	(5.390)
Bilingual x soft lockdown		− 3.417	0.775	− 0.994
		(5.254)	(4.104)	(2.051)
German x soft lockdown		11.595^∗∗∗^	13.978^∗∗∗^	7.244^∗^
		(3.725)	(2.921)	(4.145)
Observations	6,047	6,047	6,047	6,047
Adjusted R^2^	0.905	0.912	0.921	0.932
Canton + Daily FE	Yes	Yes	Yes	Yes
Health + economic controls	No	No	Yes	Yes
Region x weekly FE	No	No	No	Yes

The standard errors are wild cluster bootstrapped on cantons. ^∗^*p* < 0.1; ^∗∗^*p* < 0.05; ^∗∗∗^*p* < 0.01. Pre-lockdown and French are excluded as reference. The bilingual category includes Graubünden. Health and demographic controls: hospital beds per 1000, share of population 65+, log(population), share of urban population, population density. Socio-economic controls: trust in institutions, share of tertiary education, GDP per capita, and cross-border workers per 100,000 inhabitants, based on their country of residence (France, Germany, and Italy)

The following phase of easing measures saw the French-German mobility differences disappear while the bilingual cantons exhibited a weakly significant *higher* reduction in mobility by around 4 percentage points, suggesting that compared to the French-speaking cantons, they continued to voluntarily comply with social distancing measures. In general, however, over the course of the pandemic, we do not find strongly significant differences for the bilingual group and this may be precisely due to the inter-cantonal asymmetries providing an interesting middle ground. While Fribourg and Valais have a larger share of French speakers, Bern and Graubünden are majority German speakers.

**Fig. 5:**
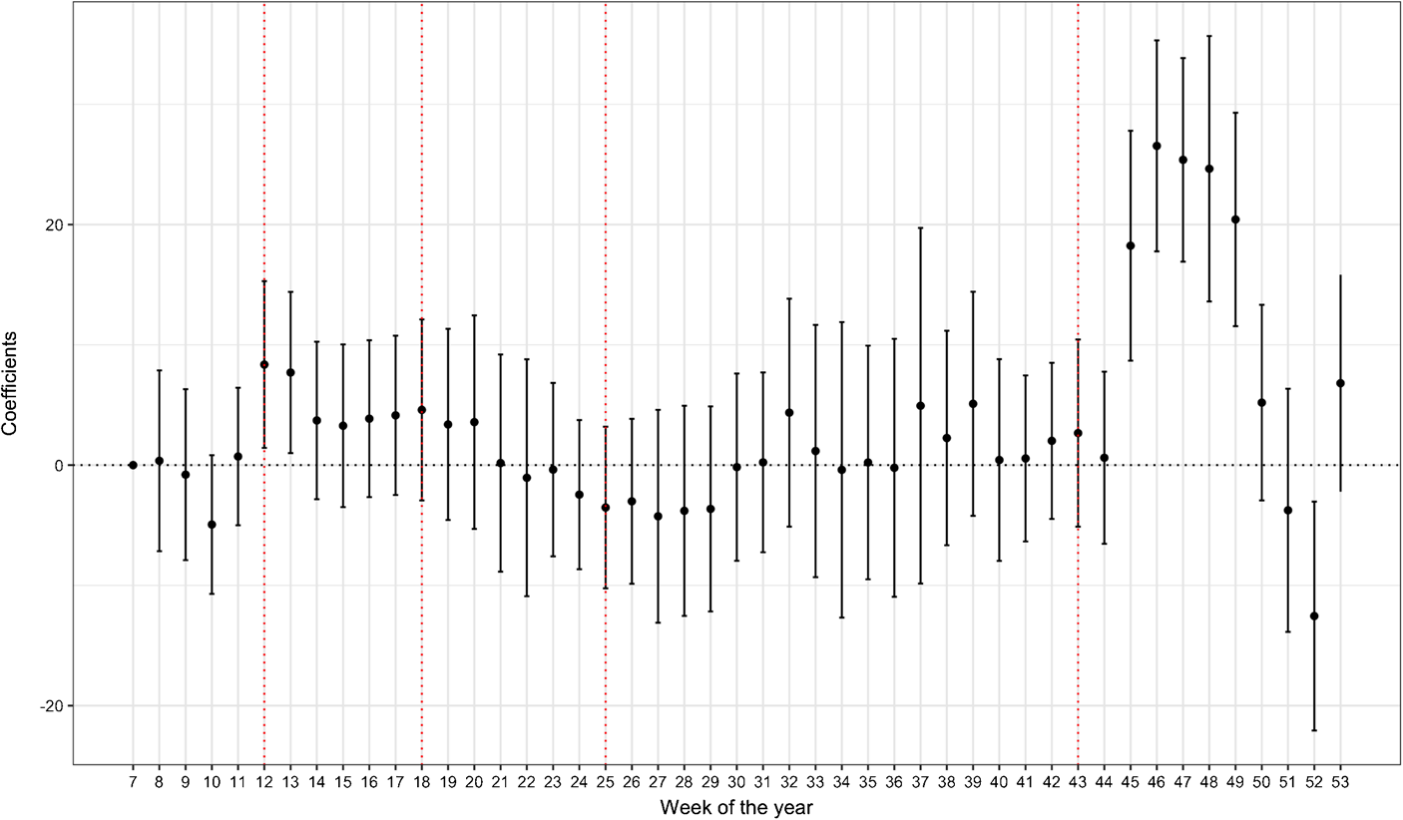
Difference in mobility between German- and French-speaking cantons. The national lockdown was implemented on the first day of Week 12 (March 16 - March 22)

During the soft lockdown implemented to counter the second wave, the difference in mobility between German and French region becomes large and significant, as seen in columns (2) and (3). This can be explained by regulatory differences and the more restrictive stance taken by the latter group of cantons. Once we account for region×week fixed effects, thus controlling for this variation to some degree, there is considerable decline in the coefficient’s magnitude and significance. Figure 5 plots the coefficients of the interactions of language with weekly time dummies and shows the average differences in weekly mobility between the German and French cantons over several phases of the pandemic. Prior to the national lockdown, there is no significant difference between the two linguistic groups but the divergence in mobility patterns becomes significantly positive after the Federal Council’s declaration of an “extraordinary situation” and continues to remain significant up until week 13. Similar to our results, these differences seem to dissipate over the next two phases and resurface during the second lockdown.

Table 3 in column (1) explores the cultural trait of generalized trust towards others. The belief about others’ trustworthiness, which also represents a widely used measure of civism and social capital, has been associated with cooperative and altruistic behavior (Brehm and Rahn 1997; Uslaner 2002). Estimating specification (2a) we find that the interaction between high trust and the dummy for the period of national lockdown displays a positive and significant coefficient, with high trusting cantons exhibiting a 7 percentage points *lower* drop in mobility than the rest. High interpersonal trust may lead individuals in these cantons to believe that even while travelling, fellow citizens will behave responsibly by following physical distancing and hygiene rules. This belief therefore reduces the (perceived) benefit of limiting individual mobility as meeting strangers and acquaintances involves a relatively lower (perceived) risk of contracting and spreading the disease. Coherently, the subsequent phase which saw declining COVID cases and easing of measures led the positive difference in mobility to amplify due to the high trust that others will continue to follow the rules and adhere to individual responsibility.

**Table 3:** Cultural dimensions

	Dependent variable:		
	Retail & Recreation		
	(1)	(2)	(3)
New cases per capita	− 0.024^∗∗∗^	− 0.023^∗∗∗^	− 0.022^∗∗∗^
	(0.008)	(0.008)	(0.007)
New deaths per capita	− 0.651^∗∗^	− 0.588^∗∗^	− 0.563^∗∗^
	(0.297)	(0.289)	(0.269)
High trust x national lockdown	6.718^∗∗^		
	(3.340)		
High trust x easing of measures	11.659^∗∗∗^		
	(3.845)		
High trust x no restrictions	4.069		
	(4.085)		
High trust x soft lockdown	4.724		
	(5.192)		
High diminution x national lockdown		5.774^∗∗∗^	
		(1.538)	
High diminution x easing of measures		− 0.352	
		(2.380)	
High diminution x no restrictions		− 0.467	
		(2.316)	
High diminution x soft lockdown		1.414	
		(2.627)	
% German spoken x national lockdown			0.108^∗∗^
			(0.049)
% German spoken x easing of measures			− 0.011
			(0.038)
% German spoken x no restrictions			0.040
			(0.063)
% German spoken x soft lockdown			0.069^∗^
			(0.041)
Observations	6047	6047	6047
Adjusted R^2^	0.932	0.932	0.932

All specifications include canton, daily and region×week fixed effects. The standard errors are wild cluster bootstrapped on cantons. ^∗^*p* < 0.1; ^∗∗^*p* < 0.05; ^∗∗∗^*p* < 0.01. The following control variable are included—health and demographic controls: hospital beds per 1000, share of population 65+, log(population), share of urban population, population density. Socio-economic controls: trust in institutions, share of tertiary education, GDP per capita and cross-border workers per 100,000 inhabitants, based on their country of residence (France, Germany, and Italy)

Column (2) looks at preferences for redistribution which can reflect innate attitudes with respect to government intervention. It may emphasise beliefs and values about whether a state is responsible for the common good such as managing a health and economic crisis. Estimating specification (2b), we find that during the national lockdown, cantons with high share of individuals in favour of reducing social spending saw 6 percentage points *lower* decline in mobility than the rest. This could be due to their hesitancy to fully support a federal intervention as the population in these cantons is likely to be more uncomfortable with public decisions entailing severe limitations of personal liberties to preserve the social welfare. These traits are also consistent with a politically conservative affiliation. Additionally, since these cantons show low support for re-distributive policies, displaying a more individualistic culture, collective action of a coordinated pandemic response may have met with some degree of reluctance. Figure 10a and b show the average differences in weekly mobility for both these cultural indicators.

In Deopa and Forunato (2021), we show detailed results for specification (1), (2a) and (2b) with the entire set of control variables (Table A3 and A4). Predictably, we find that the presence of Italian cross-border workers is consistently associated with reduced mobility. As Italy has been an epicenter of COVID-19 in Europe and one of the most affected western countries, these results possibly reflect the anxiety diffused in the Italian population which spread over neighboring Swiss regions. We also find that although the health infrastructure i.e. the number of hospital beds per 1000 inhabitants did not have an effect on compliance during the national lockdown, it was significant and positively associated with mobility reduction in the second and third phase of the pandemic.

Geographic and demographic variables such as total population, population density and the share of population older than 65, had a negative and significant effect on mobility patterns but only since the government started easing the measures. Conversely, the share of urban population was positively correlated with mobility. Another element that may have played a role in explaining compliance to social distancing measures is trust in institutions, which is correlated with trust in others and captures the confidence in the efficiency of the government. While this variable had a sizeable effect on reducing mobility from the second phase onward, it did not seem to affect compliance to mandatory federal restrictions during the national lockdown.

### Robustness

To further confirm the important role of culture and emphasise the differences observed amongst the linguistic regions, we re-estimate specification (1) by taking a continuous measure of language which is the share of the cantonal population with German (or Swiss German) as their main language. In column (3) of Table 3, we find that the interaction between percentage of German spoken and the dummy for national lockdown has a positive and significant coefficient. Correspondingly, Fig. 11a illustrates the average marginal effects indicating that particularly during the federal council’s intervention, cantons with higher share of German as their main language displayed a *lower* decline in mobility compared to pre-lockdown.

Additionally, as a robustness check we make use of “The KOF Stringency Plus Index” which records the stringency of COVID-19 policy measures in Switzerland and in all cantons.^14^ Similar to the Oxford stringency index, it provides daily indices and illustrates the level of lockdown policies over time and between cantons. The values range from 0 (= no measures) to 100 (= full lockdown). The national measures constitute the minimal level of implementation for every canton but they can introduce stricter measures if preferred. We regress the mobility measure of retail and recreation of canton *c* at day *t* in the following different specifications:

3 $$ \begin{array}{@{}rcl@{}} m_{ct} &=& \text{S} + {\delta^{S}_{1}} \text{New cases}_{c,t-1} + {\delta^{S}_{2}} \text{New deaths}_{c,t-1} + \lambda^{S} \textbf{X}_{ct}+\alpha_{c} + \theta_{t} + \epsilon_{ct} \\ \text{where} && \\ \text{S} &=& ({\beta^{S}_{1}} + {\beta_{2}^{S}} \text{Language}_{c}) \text{Stringency}_{ct} \end{array} $$

4 $$ \begin{array}{@{}rcl@{}} \text{S} &=& ({\beta^{S}_{1}} + {\beta_{2}^{S}} \text{High trust}_{c}) \text{Stringency}_{ct} \end{array} $$

5 $$ \begin{array}{@{}rcl@{}} \text{S} &=& ({\beta^{S}_{1}} + {\beta_{2}^{S}} \text{High diminution}_{c}) \text{Stringency}_{ct} \end{array} $$

Estimates in Table 4 confirm the robustness of our previous findings. Column (1) and Fig. 11b show that the difference in mobility between German- and French- speaking cantons is significantly positive and increasing for all levels of stringency. Additionally, the difference for bilingual cantons continues to be insignificant. Also consistent with our earlier results, we find a significant and positive coefficient for high trust and high diminution interaction terms. This suggests that while stringency certainly lowered mobility, the difference in reduction for high trust and high diminution was positive and increasing, indicating a reluctance to comply with COVID-19 measures.

**Table 4:** Robustness - stringency index

	Dependent variable:		
	Retail & Recreation		
	(1)	(2)	(3)
Bilingual x stringency	− 0.026		
	(0.055)		
German x stringency	0.126^∗∗∗^		
	(0.047)		
High trust x stringency		0.083^∗∗∗^	
		(0.031)	
High diminution x stringency			0.082^∗∗∗^
			(0.012)
Stringency	− 0.906^∗∗∗^	− 0.833^∗∗∗^	− 0.868^∗∗∗^
	(0.189)	(0.199)	(0.214)
New cases per capita	− 0.035^∗∗∗^	− 0.033^∗∗∗^	− 0.033^∗∗∗^
	(0.009)	(0.010)	(0.010)
New deaths per capita	− 1.585^∗∗∗^	− 1.763^∗∗∗^	− 1.852^∗∗∗^
	(0.455)	(0.457)	(0.472)
Cross-border workers per capita (French)	− 0.021^∗^	− 0.024^∗^	− 0.024^∗∗^
	(0.013)	(0.013)	(0.012)
Cross-border workers per capita (German)	0.020	0.031	0.027
	(0.051)	(0.050)	(0.052)
Cross-border workers per capita (Italian)	− 0.032^∗∗∗^	− 0.032^∗∗∗^	− 0.032^∗∗∗^
	(0.003)	(0.003)	(0.003)
Observations	6,047	6,047	6,047
Adjusted R^2^	0.911	0.909	0.910

All specifications include canton and daily fixed effects. The standard errors are wild cluster bootstrapped on cantons. ^∗^*p* < 0.1; ^∗∗^*p* < 0.05; ^∗∗∗^*p* < 0.01. Stringency refers to The KOF Stringency Plus Index. The values range from 0 (= no measures) to 100 (= full lockdown). The bilingual category includes Graubünden

Finally, we include Ticino in our sample and replicate our main results in Table 5. Consistent with Table 2, during the national lockdown, we find the difference between French and the bilingual cantons together with Ticino continue to be insignificant. However, with this additional inclusion, during the subsequent phase of easing measures the higher reduction in mobility displayed by these group of cantons becomes stronger in significance with an approximately similar magnitude of 4 percentage points. Likewise, the estimates for the interaction of cultural dimensions of trust in others and share in favour of diminution are comparable with that of Table 3. Therefore, we can substantiate that even with the addition of Ticino, our results continue to be robust.

**Table 5:** Robustness—including Ticino

	Dependent variable:		
	Retail & Recreation		
	(1)	(2)	(3)
New cases per capita	− 0.020^∗∗∗^	− 0.022^∗∗∗^	− 0.021^∗∗∗^
	(0.008)	(0.008)	(0.008)
New deaths per capita	− 0.553^∗^	− 0.615^∗∗^	− 0.556^∗∗^
	(0.294)	(0.296)	(0.282)
(Bilingual+Ticino) x national lockdown	1.041		
	(2.821)		
German x national lockdown	7.270^∗∗^		
	(3.299)		
(Bilingual+Ticino) x easing of measures	− 4.030^∗∗^		
	(2.026)		
German x easing of measures	3.687		
	(4.776)		
(Bilingual+Ticino) x no restrictions	− 0.106		
	(3.351)		
German x no restrictions	3.770		
	(5.405)		
(Bilingual+Ticino) x soft lockdown	− 1.171		
	(2.021)		
German x soft lockdown	7.716^∗^		
	(4.565)		
High trust x national lockdown		6.839^∗∗^	
		(3.372)	
High trust x easing of measures		12.063^∗∗∗^	
		(3.877)	
High trust x no restrictions		5.121	
		(4.188)	
High trust x soft lockdown		6.374	
		(4.946)	
High diminution x national lockdown			5.881^∗∗∗^
			(1.616)
High diminution x easing of measures			− 0.090
			(2.482)
High diminution x no restrictions			− 0.166
			(2.539)
High diminution x soft lockdown			1.754
			(2.762)
Observations	6364	6364	6364
Adjusted R^2^	0.934	0.934	0.934

We include Ticino in our sample and replicate our results from specifications (1), (2a), and (2b). For linguistic classification, we combine the Bilingual cantons and Ticino as one group. We redefine the dummy for high trust and high diminution based on the distribution of the relevant sample. All specifications include canton, daily, and region×week fixed effects. The following control variable are included—health and demographic controls: hospital beds per 1000, share of population 65+, log(population), share of urban population, population density. Socio-economic controls: trust in institutions, share of tertiary education, GDP per capita and cross-border workers per 100,000 inhabitants, based on their country of residence (France, Germany, and Italy). Pre-lockdown and French are excluded as reference. The bilingual category includes Graubünden. The standard errors are wild cluster bootstrapped on cantons ^∗^*p* < 0.1; ^∗∗^*p* < 0.05; ^∗∗∗^*p* < 0.01

## Conclusion

The success of non-pharmaceutical interventions undertaken to contain the spread of COVID-19 since the early stages of the pandemic has varied greatly across and within countries. This is particularly true for restrictive measures aimed at reducing individual mobility and inducing social distancing. Focusing on Switzerland, we investigate how specific cultural traits associated with the linguistic background can shape individual mobility decisions. Our results show that mobility in the German-speaking areas of the country was generally less elastic to federal and cantonal restrictions than in French cantons. Compliance to social distancing was therefore weaker in German-speaking Switzerland throughout the pandemic. Moreover, we find that mobility reduction was less conspicuous in areas where individuals displayed high levels of trust in others and had a relatively low tolerance for state interventions in the economy.

Our results on the impact of high trust on compliance with national measures are at odds with recent work on civic capital and mobility. For example, Brodeur et al. (2021) show that high trust American counties decreased their mobility significantly more than low-trust counties post-lockdown. However, our results on the impact of low preference for redistribution are broadly consistent with the findings of Bazzi et al. (2021) and Frey et al. (2020) who show that individualism and an anti-state stance hampered the response to COVID-19. In the American context, lower compliance was also strongly associated with Republican support as the party is against government intervention on multiple fronts.

**Fig. 6:**
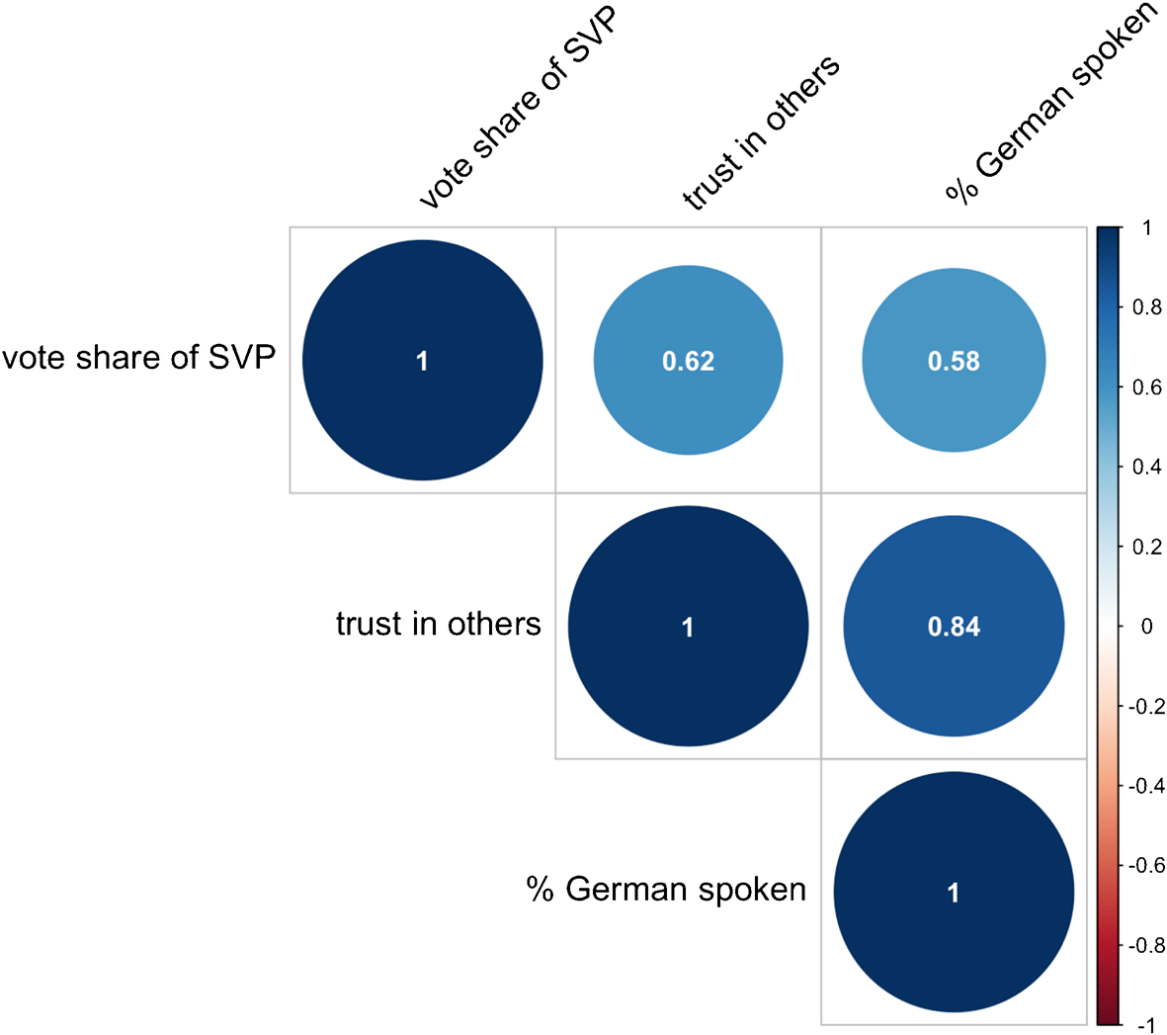
Correlation matrix

**Fig. 7:**
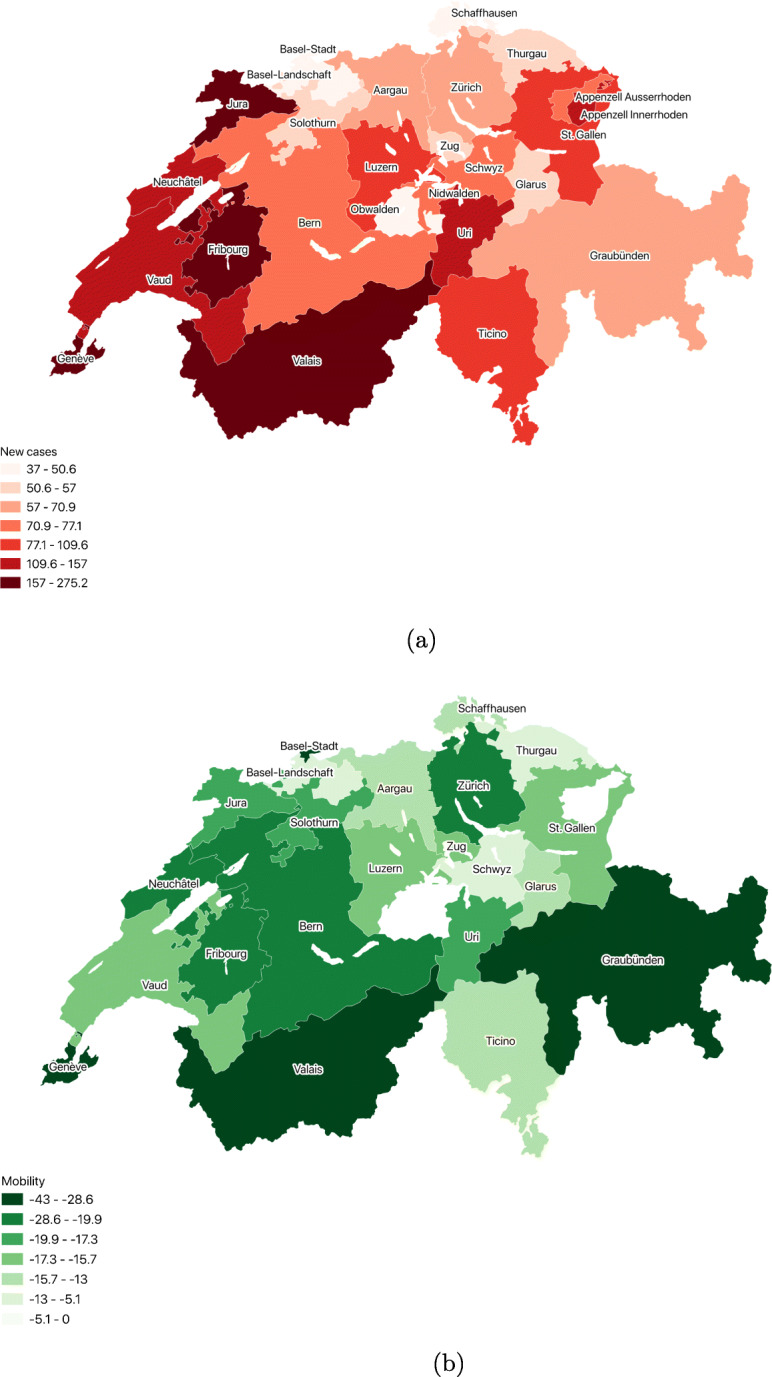
Cantonal distribution of COVID-19 cases and mobility patterns on October 30, 2020. (a) New COVID-19 cases per 100,000 inhabitants. (b) Change in mobility: Retail & Recreration

**Fig. 8:**
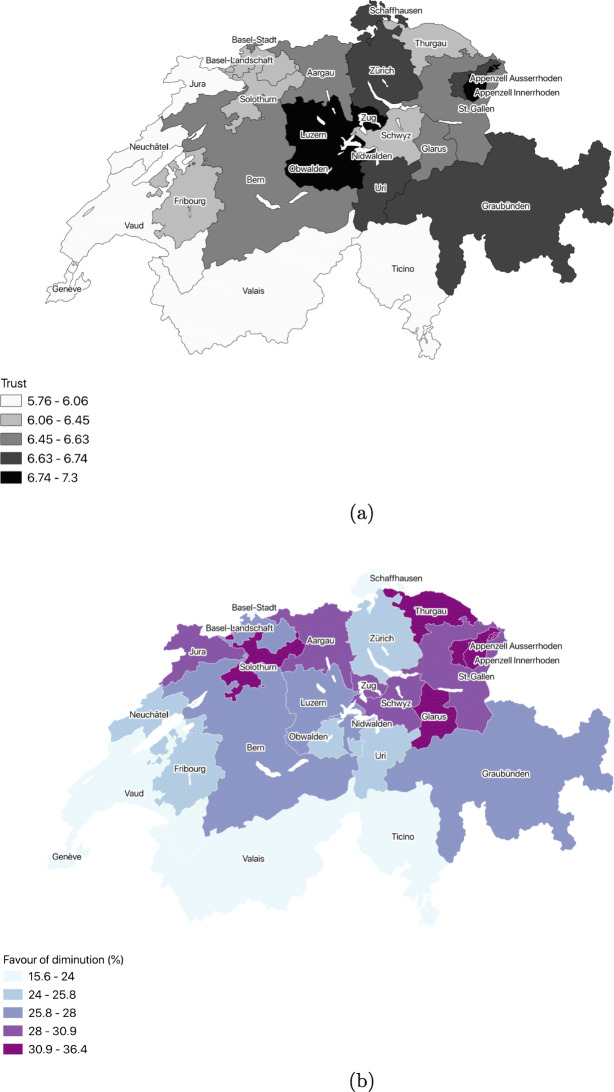
(a) Trust in others. (b) Share in favour of a diminution of Confederation social spending

**Fig. 9:**
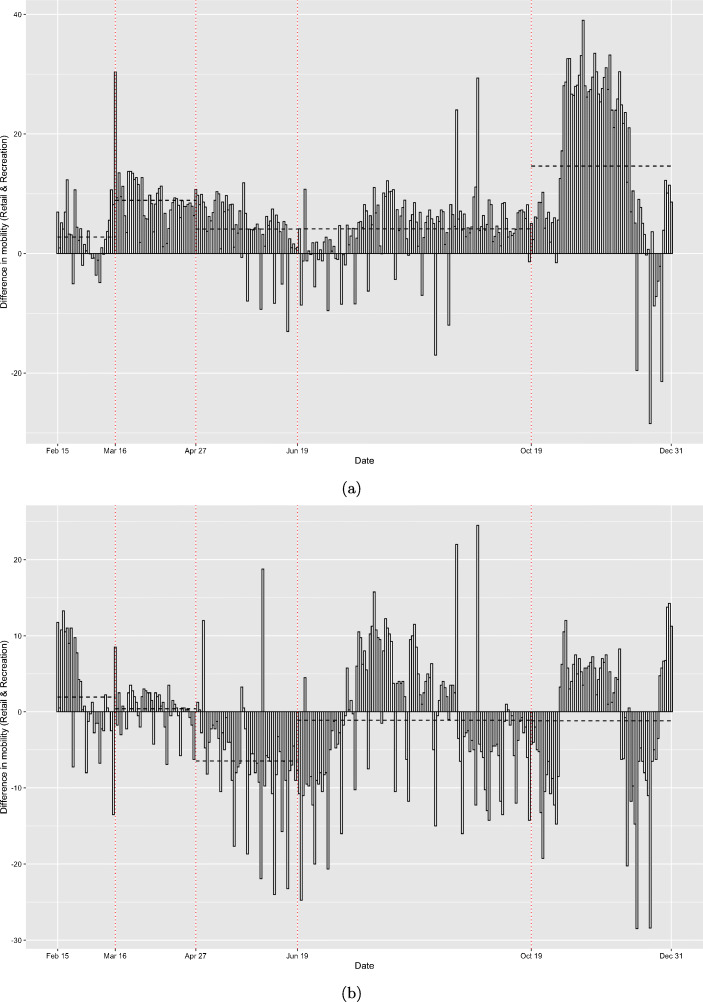
Difference in daily mobility (Retail & Recreation) using raw data. (a) German- and French-speaking cantons. (b) Bilingual and French-speaking cantons. The horizontal dashed lines are the period means

**Fig. 10:**
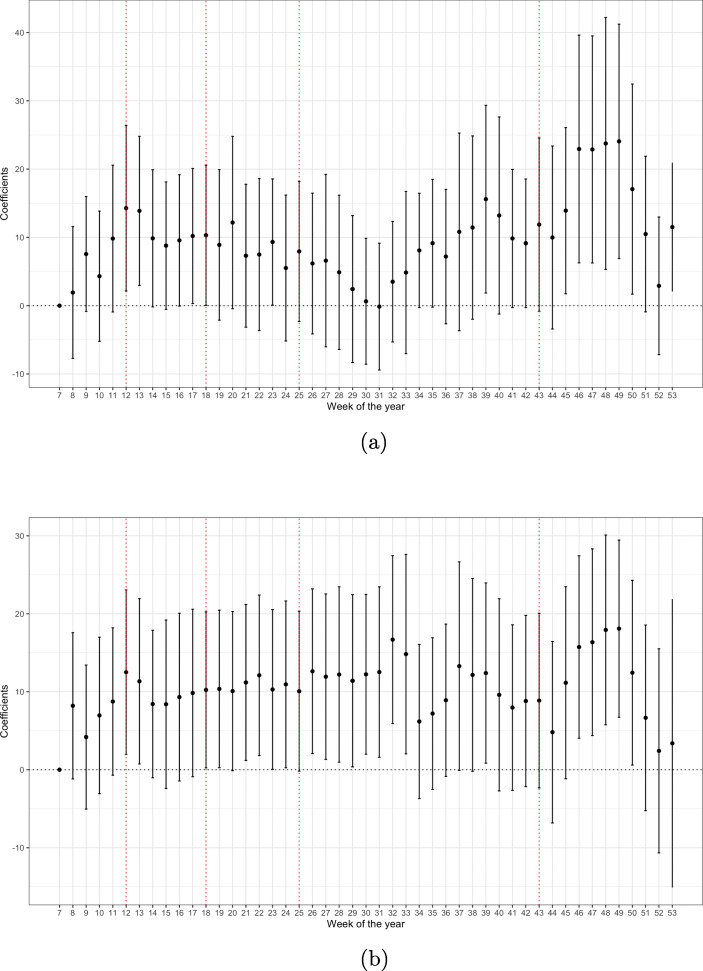
(a) Difference in mobility between high trusting cantons and rest. The national lockdown was implemented on the first day of week 12 (March 16–March 22). (b) Difference in mobility between high diminution cantons and rest. The national lockdown was implemented on the first day of week 12 (March 16–March 22)

**Fig. 11:**
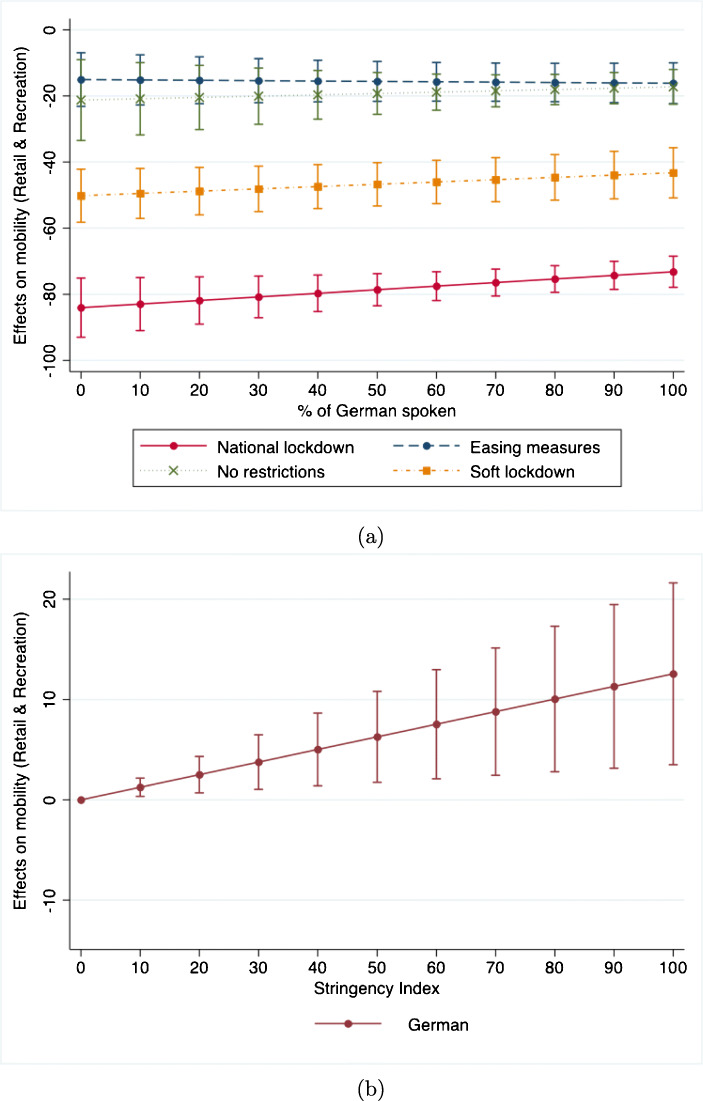
Average marginal effects

A plausible explanation for our results may be due to the unique Swiss context where the German-speaking cantons are characterized by a combination of high interpersonal trust, strong individualistic values and conservative political attitudes. It is of interest to note that many cantons within the German linguistic region are the stronghold of The Swiss People’s Party also known as the Democratic Union of the Centre (SVP/UDC), which has consistently won the largest share of votes in the national council since 1999. Ideologically the party stands for the rejection of the expansion of the welfare state, lower taxation and was extremely critical and vocal during the pandemic to reopen the economy. A simple correlation matrix between the vote share of SVP, trust in others and the percentage of German as the main language, shows these elements to be highly and positively correlated (Fig. 6), suggesting that a combination of these cultural traits may have played a role in undermining policy responses. This is also reflected in the results of a public survey where a third of the Swiss Germans declared that the measures enacted by the federal government were too extreme, against only 18% of Swiss French^15^. Overall, our results highlight how the same cultural traits may elicit different responses under a crises situation and that understanding the country specific context is crucial to policy implementation.

## Footnotes

1 see Papageorge et al. (2021), Van Bavel et al. (2020), Ding et al. (2020), and Ananyev et al. (2021)

2 see van Humboldt (1836), Mandelbaum (1951), Whorf (1956), Sapir (1968), and Boas (1982)

3 Several works of public economics and trade exploit this unique variation in languages within Switzerland (Eugster et al. 2017; Egger and Lassmann 2015; Athias and Wicht 2014).

4 This included football and ice hockey championships, carnivals in Basel and Lucerne, the Geneva Motor Show and Baselworld watch fair.

5 It allows the Federal Government to order all necessary measures for parts of or the entire country, including the right to overrule measures that were ordered by cantons and to deviate from all existing Federal laws.

6 For more information, see the following Swissinfo (international unit of the Swiss Broadcasting Corporation ) articles: “How Covid-19 is crash-testing Swiss federalism” - https://www.swissinfo.ch/eng/how-covid-19-is-crash-testing-swiss-federalism/46164134 and “Some Swiss cantons tighten Covid-19 measures, others wait” - https://www.swissinfo.ch/eng/some-swiss-cantons-tighten-covid-19-measures--others-wait/46116310

7 For a detailed timeline of the measures implemented by the Federal Office of Public Health, refer to their website: https://www.bag.admin.ch/bag/en/home/krankheiten/ausbrueche-epidemien-pandemien/aktuelle-ausbrueche-epidemien/novel-cov/massnahmen-des-bundes.html

8 For more information, see the Radio Télévision Suisse (RTS) article - “Le mystérieux “Coronagraben” au sein même des cantons bilingues” - https://www.rts.ch/info/suisse/11755389-le-mysterieux-coronagraben-au-sein-meme-des-cantons-bilingues.html

9 https://www.google.com/covid19/mobility/

10 Within the bilingual category, we also include Graubünden which is technically trilingual.

11 Main language is defined as one in which individuals think or speak best, according to their indication.

12 We use different waves as not all modules are repeated annually.

13 These are based on the FSO statistics of cross-border workers in the 4th quarter of 2018

14 This data has been made freely available by KOF Swiss Economic Institute at https://kof.ethz.ch/en/forecasts-and-indicators/indicators/kof-stringency-index.html

15 This survey was carried out by Sotomo research institute and more information can be found at https://www.rts.ch/info/suisse/11314737--coronagraben-quand-romands-et-alemaniques-ne-vivent-pas-la-meme-crise.html
